# Vaccines and immunization strategies for dengue prevention

**DOI:** 10.1038/emi.2016.74

**Published:** 2016-07-20

**Authors:** Yang Liu, Jianying Liu, Gong Cheng

**Affiliations:** 1 Tsinghua-Peking Center for Life Sciences, School of Medicine, Tsinghua University, Beijing 100084, China; 2 School of Life Science, Tsinghua University, Beijing 100084, China

**Keywords:** dengue virus, mosquito-based immunization strategies, NS1-based immunization strategies, vaccine

## Abstract

Dengue is currently the most significant arboviral disease afflicting tropical and sub-tropical countries worldwide. Dengue vaccines, such as the multivalent attenuated, chimeric, DNA and inactivated vaccines, have been developed to prevent dengue infection in humans, and they function predominantly by stimulating immune responses against the dengue virus (DENV) envelope (E) and nonstructural-1 proteins (NS1). Of these vaccines, a live attenuated chimeric tetravalent DENV vaccine developed by Sanofi Pasteur has been licensed in several countries. However, this vaccine renders only partial protection against the DENV2 infection and is associated with an unexplained increased incidence of hospitalization for severe dengue disease among children younger than nine years old. In addition to the virus-based vaccines, several mosquito-based dengue immunization strategies have been developed to interrupt the vector competence and effectively reduce the number of infected mosquito vectors, thus controlling the transmission of DENV in nature. Here we summarize the recent progress in the development of dengue vaccines and novel immunization strategies and propose some prospective vaccine strategies for disease prevention in the future.

## DENGUE HISTORY, EPIDEMIOLOGY AND ETIOLOGY

Dengue fever is a typical mosquito-borne disease with global importance for public health. The history of dengue epidemics has been recorded since the late 1800s. The first clinical case of dengue infection was diagnosed in 1789 by Benjamin Rush, a physician and one of the United States' Founding Fathers, who characterized the disease as a ‘bilious remitting fever'.^1^ The term dengue fever (DF) was generally used as a standard name for the disease after 1828.^2^ South America, Africa and Southeast Asia are the original dengue endemic regions. Over the past 200 years, however, burgeoning international travel and expanded urbanization have increased the prevalence of dengue in new territories. Dengue fever has rapidly spread within countries and across regions, which has resulted in an increased frequency of epidemics and severe dengue disease across the globe.^3^ Currently, DF has become the most widely spread arthropod-borne viral disease afflicting tropical and sub-tropical countries worldwide.

Dengue is one of the most common infectious diseases, and it is endemic in more than 110 countries.^4^ In recent decades, DF has spread to Southern China, countries in the Pacific ocean and America.^5^ In addition, it represents a potential threat to Europe.^6^ It is estimated that 390 million people are infected with dengue annually, 96 million of whom manifest overt clinical symptoms and require hospitalization.^7^ These hospitalizations result in ~25 000 deaths.^8^ In mainland China, a number of large dengue outbreaks has been reported since 1978. From 1978 to 2008, more than 600 000 total dengue cases were reported.^9^ Several southern provinces, such as Guangdong, Hainan, Yunnan and Guangxi, are predominant endemic areas of dengue. From 1990 to 2010, there have been no apparent dengue epidemics in China (>10 000 cases). However, frequent small outbreaks (1000–7000 cases) ensure long-term viral circulation in local regions.^10^ A local outbreak in the Guangdong and Yunnan provinces caused more than 50 000 infected cases in 2013–2014, and severe hemorrhagic fever developed in hundreds of these patients. Among these cases, six deaths were reported in the 2014 outbreak.^11^

Dengue in humans presents with a broad spectrum of clinical symptoms and signs that can range from mild fever (DF) to dengue hemorrhagic fever. The latter can progress to dengue shock syndrome and death.^12^ The dengue virus (DENV), which belongs to the Flaviviridae family, is the etiologic agent responsible for dengue disease. The genome of the DENV is composed of a single positive-stranded RNA that encodes three structural proteins (capsid protein C, pre-membrane protein prM and envelope protein E) and seven nonstructural proteins (NS1, NS2a, NS2b, NS3, NS4a, NS4b and NS5). The DENV E protein, a surface protein of virions, includes the main epitopes for the generation of neutralizing antibody. Hence, it is considered an optimal target for vaccine development. Moreover, the DENV NS1 protein is abundantly secreted from infected host cells and circulates in the patient's sera.^12, 13^ As a virus-encoded extracellular component, NS1 is the other potential vaccine candidate against the DENV infection.

## DENGUE LIFE CYCLE BETWEEN HUMANS AND MOSQUITOES

The DENV is generally transmitted through two separate life cycles, i.e., sylvatic and urban cycles, among mosquitoes, humans and primates.^14^ Of these hosts, humans and lower primates are the only mammalian reservoirs for dengue transmission. Four serotypes of DENV (DENV1–DENV4) that are transmitted by mosquitoes have been shown to cause human infections in the urban cycle. The viruses are acquired by mosquitoes feeding on an infected human. Subsequently, the viruses infect the mosquito midgut epithelial cells and spread systemically through the hemocoel to the salivary glands and other tissues. At that point, the infected mosquitoes are ready to transmit the virus to an uninfected human through their bites.^15^ In the urban cycle, DENV is primarily transmitted to humans via bites from the mosquitoes *Aedes aegypti* and *Aedes albopictus*. As an anthropophilic mosquito in and around human dwellings, *A. aegypti* is readily susceptible to DENV and is the primary vector for DENV transmission in tropical and sub-tropical areas. A related mosquito species, the Asian tiger mosquito, *A. albopictus*, is the secondary DENV vector. Because of its capacity for survival in temperate regions, *A. albopictus* has recently and rapidly invaded all continents, except Antarctica. As a result, *A. albopictus* is capable of causing arboviral diseases, including DENV, in areas where they are currently absent. Indeed, *A. albopictus* is a major vector for DENV transmission in China and Europe.^5, 6, 16^

## LICENSED DENGUE VACCINE

Vaccines are a principal preventive approach for combating infectious diseases. Owing to the simultaneous prevalence of four different serotypes, a practical dengue vaccine should provide long-term protection for infections of homotypic and heterotypic serotypes. Notably, a tetravalent dengue vaccine (Dengvaxia), developed by Sanofi Pasteur (Lyon, France), has been granted a marketing authorization in several countries such as Mexico and the Philippines for use in clinical practices.^17, 18^ The Dengvaxia vaccine is the first licensed vaccine in the world for dengue prevention.^18^

The Dengvaxia vaccine is a tetravalent chimeric vaccine. For each of the four dengue serotypes, the *prM* and *E* genes from virulent DENV strains are substituted into the backbone of the yellow fever virus 17D vaccine strain.^19, 20^ A tetravalent mixture of the monovalent chimeric virus was used for clinical assessment. The tetravalent vaccine is genetically and phenotypically stable and, in preclinical and phase I studies, appeared safe with relatively low viremia.^21, 22^ A phase IIb study in Thailand found this vaccine to be highly effective against DENV3 and DENV4 serotypes, with modest protection against DENV1. However, it offered almost no protection against DENV2 infection.^23^ Recently, two large-scale phase III efficacy trials have been conducted in endemic areas of Latin America and Asia. The efficacy of the serotype-specific vaccine was 50.3% for DENV1, 42.3% for DENV2, 74.0% for DENV3 and 77.7% for DENV4 in five dengue-endemic Latin American countries.^24^ In the Asia-Pacific region, the estimated average vaccine efficacy is 56.5%, with its greatest impact being in the prevention of severe dengue-related clinical symptoms and hospitalization.^25^ Similar to the phase IIb study in Thailand, the serotype-specific efficacy of the vaccine for DENV2 was not statistically significant.^25^ In addition, a recent long-term follow-up study of 35 000 children between the ages of 2 and 16 years old in Asia-Pacific and Latin American countries reported an unexplained increased incidence of hospitalization for severe dengue disease among children younger than 9 years old.^17^ These results indicate that the efficacy and safety of the Dengvaxia vaccine require further evaluation.^26^

## DENGUE VACCINES UNDER PRECLINICAL AND CLINICAL TRIALS

In addition to the licensed Dengvaxia vaccine, several dengue vaccine candidates are in clinical trials or under preclinical evaluation, and multiple strategies have been exploited for vaccine development.

### Live attenuated dengue vaccines

Live attenuated vaccines, which contain attenuated pathogenic microorganisms, are capable of producing a broad range of immune responses. However, they do not cause significant pathological sequelae. Indeed, the vaccine strategy of virus attenuation has been successfully utilized in the development of multiple flavivirus vaccines, such as the Japanese encephalitis virus SA-14-14-2 and the yellow fever virus 17D attenuated vaccines, respectively.^27, 28^ Nevertheless, the development of DENV attenuated vaccines has still not been successful in disease prevention. Several of these vaccine candidates are undergoing clinical trials.

A tetravalent attenuated dengue vaccine (LAV), developed by the scientists of Mahidol University in Thailand, was generated by serial passaging of four DENV serotypes in a cell culture. Three dengue serotype viruses (DENV1, 2 and 4) were attenuated in primary dog kidney cells, whereas DENV3 was serially passaged to reduce its virulence in primary African green monkey kidney cells.^29, 30^ The candidate vaccine was used in phase I and II clinical trials in Thai adults and children. Not all of the volunteers developed antibodies for all four dengue serotypes, and some experienced unacceptable reactogenicity. Consequently, further clinical testing was terminated.^31, 32, 33^ Recently, an advanced formulation named TDEN-F17/F19 based on the above-mentioned live attenuated viruses was developed for a new cycle of clinical assessment and exhibited sufficient safety and immunogenicity in two phase II clinical studies.^34, 35^ In addition to virus attenuation by serial passaging, the virulence of these new vaccine strains was reduced by manipulation of their viral genome.^35^

A vaccine developed by scientists at the National Institute of Allergy and Infectious Diseases contains the four dengue attenuated viruses, of which the DENV1, DENV3 and DENV4 attenuated strains were developed from a genomic frame of the wild-type viruses with a deletion in the 3′- untranslated region.^36^ The DEVN2 attenuated strain was a chimeric virus with a replacement of the DENV2 *prM* and *E* genes into the backbone of the DENV4 attenuated virus.^37^ These DENV attenuated strains have been individually tested for attenuation and immunogenicity in animal models and humans. Monovalent attenuated viruses were combined into different tetravalent mixtures (TV001–TV005) to assess the immunogenicity and safety. TV003, a candidate of tetravalent combination of attenuated viruses, is currently being tested in a phase II clinical trial.^38, 39^

### Live chimeric dengue vaccines

In addition to the licensed Dengvaxia vaccine, a tetravalent dengue vaccine candidate (TDV), developed by Takeda, is a chimeric dengue vaccine that contains a mixture of attenuated DENV2 and chimeric DENV1, DENV3 and DENV4 generated from the backbone of the attenuated DENV2.^40^ The chimeric TDV strains are created by substituting three wild-type DENVs *prM* and *E* genes into the DENV2 backbone.^40^ The safety and immunogenicity of TDV have been demonstrated through independent phase I trials in the United States and in a dengue non-endemic region of Colombia.^41^ Further evaluation in a phase II clinical study is still in process in Asia and Latin America.^42^

### Inactivated dengue vaccines

Compared with the live dengue vaccines, the tetravalent inactivated vaccine is safer and elicits immune responses that are easy to balance against the four serotypes after immunization. However, the development of an inactivated dengue vaccine is impeded by a low immunogenicity and the lack of immune responses to NS proteins. Dengue virus purified inactivated vaccine (TDEN PIV) is a tetravalent purified inactivated vaccine that is currently being evaluated jointly by Glaxo Smith Kline (London, UK) and the Walter Reed Army Institute of Research (Silver Spring, MD, USA), and is being tested in a phase I clinical study in the United States. Indeed, the immunogenicity of TDEN PIV was strengthened by certain adjuvants to meet the requirement of long-term protection. Evaluation of the safety and immunogenicity of TDEN PIV in Puerto Rican adults has been scheduled to begin by the end of 2016.^43^

### Recombinant protein, DNA and subunit dengue vaccines

The DENV E protein, a surface protein with the main epitopes for neutralizing antibody generation, is the major target antigen for the development of recombinant protein, DNA and subunit dengue vaccines.^44^ The extracellular region of the DENV E protein forms an ectodomain. The ectodomain consists of three domains that are referred to as envelope protein domains I–III (EDI, EDII and EDIII). The EDIII domain includes most of the epitopes for the generation of neutralizing antibody. It has been demonstrated that immunization with recombinant EDIII can generate protective antibodies against DENV in both mouse and nonhuman primate models.^45^ A tetravalent vaccine with a consensus of recombinant peptides including four DENV EDIII domains was effective in neutralizing the four serotypes of DENV and in inducing memory immune responses.^46, 47, 48^ Conversely, EDIII-specific antibodies did not constitute a major part in the dengue antibodies in patients' sera and only contributed to a small proportion of the DENV neutralization *in vitro*.^49, 50^ Hence, the efficacy of this vaccine needs to be further validated.

The V180 is an E-based recombinant subunit vaccine developed by Merck (Darmstadt, Germany), the antigens of which are expressed via the *Drosophila* S2 cell expression system. This vaccine is currently in a phase I trial to test its safety and immunogenicity both with and without adjuvants.^51^ In addition, the US Naval Medical Research Center has developed a tetravalent DNA vaccine candidate, which is composed of equal parts of monovalent plasmid DNA encoding the *prM* and *E* genes of four DENV serotypes,^52, 53, 54, 55^ with the proprietary adjuvant Vaxfectin. This tetravalent DNA vaccine has recently cleared the phase I study, and work is ongoing to improve immunogenicity with different routes of administration and other approaches.

## NOVEL IMMUNIZATION STRATEGIES FOR DENGUE PREVENTION

Although many vaccines that target either the DENV virions or the viral structural proteins have been licensed or under clinical trials, significant safety concerns remain unresolved to date. Several concerns in dengue pathogenesis, such as the risk of antibody-dependent enhancement effect and pathogenesis caused by cross-reactive T cells during dengue infection, have raised challenges to the development of a safe and effective dengue vaccine using conventional strategies.^56, 57^ Indeed, in addition to the traditional concepts used for development of dengue vaccine, some specific properties of DENV replication and life cycle might provide interrupting targets for novel vaccine designs. Recently, several novel concepts, such as the DENV NS1-based and mosquito-based immunization strategies, have been proposed as candidates for dengue prevention in the future.

### Nonstructural protein 1 (NS1)-based dengue immunization strategies

The NS1 is a glycoprotein with three forms in DENV-infected cells: an endoplasmic reticulum-binding dimer; a membrane glycosylphosphatidylinositol anchored form; and a secreted hexamer.^58^ The secretion of the NS1 protein in patient sera reaches high levels that range from 70 to 15 000 ng/mL. In exceptional cases, this level can reach 50 000 ng/mL.^12, 13^ As a virus-encoded extracellular component, the NS1 is the other potential vaccine candidate against the DENV infections. One study reported that passive immunization with anti-NS1 antibodies prevented the DENV lethal disease in a mouse model.^59^ Moreover, either active intraperitoneal or subcutaneous immunization with recombinant NS1 protein resulted in partial protection in mice cranially challenged with DENV.^60, 61^ Two recent studies in interferon-alpha receptor-deficient mice found that the DENV NS1 protein acts as a key effector that enhances the permeability of blood vessels.^62, 63^ Therefore, NS1 immunization fully prevents dengue-mediated vascular leakage and protects interferon receptor-deficient mice from the lethal effects of DENV2 challenge.^62^ In addition to NS1 immunization, another promising strategy is to immunize with a fusion protein that includes E, NS1 and other immunogenic proteins.^64, 65^ The immunization of a fusion protein that contains the DENV2 E, the N terminal of NS1 and staphylococcal protein A efficiently generated anti-DENV2 antibodies and protected mice against a DENV2 lethal infection.^64^ Immunization with combined recombinant plasmids that expressed DENV-encoded components and inflammatory cytokines, such as the DENV prM, E, NS1 and rat granulocyte-macrophage colony-stimulating factor, induced specific anti-DENV2 immune responses and partially protected mice from a lethal intracerebral DENV2 challenge.^65^

A recombinant DNA plasmid is another potential strategy for the development of an NS1-based vaccine. The intramuscular inoculation of a *DENV2 NS1*-containing recombinant plasmid pcTRANS1 protected against a lethal DENV2 challenge by intracerebral inoculation in mice.^66^ Similarly, the immunization of plasmids that express the DENV2 NS1 elicited both NS1-specific humoral and cellular immune responses in mice. After an intravenous challenge with lethal DENV2, the immunized mice exhibited a delay in the onset of paralysis and morbidity symptoms, and they had a significantly prolonged survival.^67^ In addition, recombinant vaccine viruses that encode DENV2 or DENV4 NS1 proteins have demonstrated partial protection in BALB/c mice that were intracerebrally challenged with lethal DENV2 or DENV4.^68^ In contrast, several studies suggest that NS1 antibodies facilitate the dengue pathological sequelae due to its cross-reactivity with host proteins, thus causing the inhibition of platelet aggregation and apoptosis of endothelial cells.^69, 70^ The inoculation of certain NS1 monoclonal antibodies increased morbidity in DENV-infected mice.^59^ Clearly, the safety and efficacy of the NS1 immunization requires further evaluation.

### Transmission-blocking immunization strategies for dengue prevention

For vector-borne diseases, the stages of the life cycles of pathogens can be targeted by designing vaccines that enable the control of the diseases in the environment. The susceptibility factors that facilitate a pathogen's invasion can potentially be explored as targets for disrupting the path by which microbes invade the vertebrate or invertebrate host. A transmission-blocking immunization strategy that targets the gametocytes of the malaria pathogen, *Plasmodium spp.*, successfully reduces the number of parasites that survive in the mosquito gut and, consequently, impairs the efficiency of microbial acquisition and mosquito infectivity.^71, 72^ In conjunction with antigens that are of particular importance in the life cycles of pathogens, the host humoral immunity against the tick receptor for OspA (TROSPA), a tick gut receptor for the agent that causes Lyme disease, limits the colonization of ticks by *Borrelia burgdorferi*.^73^ A recombinant *Rhipicephalus appendiculatus* tick protein, a truncated recombinant forms of 64P (64TRP), functions as a candidate for a transmission-blocking immunization strategy to protect immunized mice against a lethal challenge with the tick-borne encephalitis virus after exposure to infected ticks.^74, 75^ Therefore, strategies that target the life cycle of DENV constitute feasible approaches to reduce the microbial dissemination.

Given the rapid increase in the spread of dengue and the disease burden over the last decade, additional strategies are urgently needed to combat the dengue dissemination.^76^ The survival of DENV is restricted to its interaction between humans and the *Aedes* mosquito. The host specificity of the dengue infection implies that vaccination of the human population is a feasible method for reducing the number of infected vectors and, consequentially, reduce the disease burden. Multiple mosquito C-type lectins (mosGCTLs) have been identified that interact with the DENV2 virus to enhance viral infection in *A. aegypti*.^77, 78^ Compared with a treatment with pre-immune sera, the combination of antisera against multiple mosGCTLs markedly reduced dengue infection after a membrane blood meal, suggesting that mosGCTL immunization in humans may help interrupt the life cycle of DENV in nature.^78^ Moreover, Colpitts and her colleagues identified a putative cysteine-rich venom protein (CRVP379) that acts as an important susceptibility factor that facilitates the DENV infection in *A. aegypti*. Blocking the CRVP379 protein with either RNAi or specific antisera inhibited the DENV infection in *A. aegypti*, representing a broad preventive and therapeutic measure for dengue control.^79^ Taken together, immunization with DENV-related mosquito susceptibility factors may also be a feasible transmission-blocking vaccine approach for dengue prevention.

## PERSPECTIVE

Dengue is currently the most significant arboviral disease afflicting tropical and sub-tropical countries worldwide. The conventional vaccine strategies, such as the multivalent attenuated, chimeric, DNA and inactivated vaccines, have been developed for the prevention of infection from all four dengue serotypes in humans, predominantly by stimulating immunity against either the DENV virions or the envelope proteins exposed on the viral surface (Table 1). However, current evidence from clinical trials indicates that these vaccines merely render partial protection against the DENV infection in humans. A live attenuated tetravalent DENV vaccine developed by Sanofi Pasteur, which has been licensed in several countries, has a low efficacy against the DENV2 infection, which is a major concern in clinical practice. Furthermore, the special mechanisms underlying dengue pathogenesis that contribute to the risk of antibody-dependent enhancement effect and the pathological responses caused by cross-reactive T cells, have raised other safety concerns for the conventional vaccines. In addition to the vaccine strategies that target the DENV virions or the envelope proteins, previous studies have indicated that immunization with NS1 protein, a DENV NS protein that is secreted into the extracellular milieu, was effective in preventing DENV-induced vascular leakage and severe clinical symptoms. The NS1-based immunization, which avoids the antibody-dependent enhancement and cross-reactive T cell-related complications caused by conventional vaccine strategies, may be an ideal candidate for use in the development of a novel dengue vaccine in the future. Moreover, the DENV life cycle offers multiple targets for a vaccine to prevent human infection or interrupt mosquito transmission. Several mosquito-based dengue immunization strategies have also been developed to interrupt the vector competence to effectively reduce the number of infected mosquito vectors and, thus, control the transmission of DENV in nature (Table 1).

Given the inadequate efficacy of conventional dengue vaccines, we propose that prospective vaccine development should combine novel vaccine concepts, such as NS1-based and mosquito-based immunization, with established vaccines to improve their safety and efficacy against dengue infection in humans and reduce transmission by mosquitoes, thereby efficiently controlling the existence of DENV in nature.

## Figures and Tables

**Table 1:** Vaccine strategies for dengue prevention

**Vaccine strategy**		**Vaccine name**	**Target antigen**	**Current stage**	**References**
Licensed	Live chimeric vaccine	Dengvaxia	prM and E	Licensure	^17, 18, 19, 20, 21, 22, 23, 24, 25, 26^
Under preclinical and clinical trials	Live attenuated vaccines	LAV	Live virus	Phase II (abandoned)	^29, 30, 31, 32, 33^
		TDEN-F17/F19	Live virus	Phase II	^34, 35^
		TV003	Live virus	Phase II	^36, 37, 38, 39^
	Live chimeric vaccine	TDV	prM and E	Phase II	^40, 41, 42^
	Inactivated vaccine	TDEN PIV	Inactive virus	Phase I	^43^
	Recombinant vaccines	EDIII-based vaccine	EDIII domain	Preclinical	^45, 46, 47, 48, 49, 50^
		V180	80% E	Phase I	^51^
		TVDV	prM and E	Phase I	^52, 53, 54, 55^
Novel immunization strategies	NS1-based immunization strategies	NS1 protein-based strategy	NS1	Discovery	^59, 60, 61, 62, 63^
		NS1 fusion protein-based strategy	E and NS1	Discovery	^64, 65^
		NS1 plasmid-based strategy	NS1	Discovery	^66, 67, 68^
	Transmission-blocking immunization strategies	C-type lectin-based strategy	Mosquito C-type lectins	Discovery	^78^
		CRVP379-based strategy	CRVP379	Discovery	^79^

Abbreviations: nonstructural-1 protein, NS1; tetravalent dengue vaccine, TDV; tetravalent DNA vaccine, TVDV.
